# The Engaged Health Sciences Library Liaison

**DOI:** 10.5195/jmla.2021.1132

**Published:** 2021-01-01

**Authors:** Angela Barr

**Affiliations:** 1 ab3538@georgetown.edu, Dahlgren Memorial Library, Georgetown University School of Medicine, Washington, DC

*The Engaged Health Sciences Library Liaison* assembles case studies from US and Canadian academic and medical institutions that highlight the unique yet diverse roles and responsibilities of a health sciences library liaison. Each case study showcases a library's innovative approach to customizing liaison services to patron needs and leveraging liaison staff as stakeholders in, and contributors to, curriculum planning and execution. This text helps early career librarians explore the options available to them in the specialty of health sciences librarianship and is ideally suited for experienced health sciences librarians or administrators who are seeking to expand the breadth and depth of services that their libraries and institutions offer.

The text is tight but readable, and each case study provides reference lists for additional reading opportunities. Figures interspersed throughout the text are a welcome addition, but some are too small to be legible (p. 33 and p. 103). The index does a good job identifying the libraries and organizations that are represented in the case studies, though readers might appreciate more expansive subject indexing.

Just as health sciences libraries serve an assorted, yet specialized patronage, *The Engaged Health Sciences Library Liaison* offers a variety of approaches and best practices to a targeted audience. Whether describing high school enrichment programs (chapter 2), undergraduate and graduate medical student instruction (chapters 3, 4, and 5), systematic review research support (chapter 6), or resident boot camps and clinical rounding (chapter 11), *The Engaged Health Sciences Library Liaison* demonstrates that health sciences library liaisons have the potential to impact decades of student years, health research, and clinical practice.

Unlike the 2015 Association of Research Libraries *Evolution of Library Liaisons,* SPEC kit 349 [1], which employs a case study structure to explore library liaison roles across academia, or the 2014 *Health Sciences Librarianship* by M. Sandra Wood, FMLA [2], which supplies a comprehensive overview of health sciences libraries, *The Engaged Health Sciences Library Liaison* focuses on its subject matter, revealing facets of health sciences library liaisons and their work that readers will find invaluable and inspiring.
